# Therapeutic Applications of Plant-Derived Extracellular Vesicles as Antioxidants for Oxidative Stress-Related Diseases

**DOI:** 10.3390/antiox12061286

**Published:** 2023-06-16

**Authors:** Manho Kim, Hyejun Jang, Wijin Kim, Doyeon Kim, Ju Hyun Park

**Affiliations:** Department of Biomedical Science, Kangwon National University, Chuncheon-si 24341, Republic of Korea; manhokim@kangwon.ac.kr (M.K.); hyejunjang@kangwon.ac.kr (H.J.); dnlwls018@kangwon.ac.kr (W.K.); rlaehdus6321@kangwon.ac.kr (D.K.)

**Keywords:** antioxidants, carcinogenesis, chronic wound healing, oxidative stress, plant-derived extracellular vesicle, skin aging

## Abstract

Extracellular vesicles (EVs) composed of a lipid bilayer are released from various cell types, including animals, plants, and microorganisms, and serve as important mediators of cell-to-cell communication. EVs can perform a variety of biological functions through the delivery of bioactive molecules, such as nucleic acids, lipids, and proteins, and can also be utilized as carriers for drug delivery. However, the low productivity and high cost of mammalian-derived EVs (MDEVs) are major barriers to their practical clinical application where large-scale production is essential. Recently, there has been growing interest in plant-derived EVs (PDEVs) that can produce large amounts of electricity at a low cost. In particular, PDEVs contain plant-derived bioactive molecules such as antioxidants, which are used as therapeutic agents to treat various diseases. In this review, we discuss the composition and characteristics of PDEVs and the appropriate methods for their isolation. We also discuss the potential use of PDEVs containing various plant-derived antioxidants as replacements for conventional antioxidants.

## 1. Introduction

Oxidative stress is defined as an imbalance between reactive oxygen species (ROS) generation and the antioxidant defense systems [1]. ROS are bioactive molecules that are metabolites of the respiratory processes of living organisms. Various environmental stresses can lead to the excessive generation of ROS [2,3]. Excessively generated ROS can cause damage to cell membranes as well as DNA and proteins, leading to cell death, which can cause a variety of pathological problems [4]. Unlike animals, plants are immobile and therefore cannot escape various environmental stresses [5]. Environmental stressors such as ultraviolet (UV) light, salinity, temperature extremes, drought, and pathogens can trigger ROS generation in plants, and the excessive accumulation of ROS can lead to oxidative stress [6]. Accordingly, plants have evolved antioxidant defense systems to protect themselves from the oxidative stress caused by excessive ROS generation [7,8].

Plant antioxidant defense systems can be divided into enzymatic and non-enzymatic antioxidants. Enzymatic antioxidants, such as superoxide dismutase (SOD), glutathione peroxidase (GPX), catalase (CAT), and glutathione reductase, directly remove a variety of toxic oxidants from multiple sites, including chloroplasts and cytosol [9]. Plants contain many different types of antioxidants, including non-enzymatic phytochemicals, depending on the type and origin of the plant [10,11]. Vitamin C, phenolic compounds, and carotenoids are major plant-derived antioxidative phytochemicals [12,13]. These plant-derived antioxidative phytochemicals have long been extracted using a variety of methods and utilized to treat diseases caused by oxidative stress, including chronic wounds, carcinogenesis, and skin aging [14,15,16]. Plant-derived extracellular vesicles (PDEVs) containing various plant-derived phytochemicals, particularly plant-derived antioxidants, have recently gained prominence. In this review, we provide a comprehensive overview of the composition and properties of PDEVs and their isolation methods. We further discuss the potential applications of PDEVs as alternatives to traditional antioxidants to address various pathological problems caused by oxidative stress.

## 2. Oxidative Stress Induced by ROS

### 2.1. Oxidative Stress

Aerobic cellular respiration, which occurs in the mitochondria of eukaryotic cells, generates energy, and oxidative metabolism produces several compounds. Although these metabolites are mostly beneficial, excessive metabolite levels can lead to toxicity [17]. ROS are natural byproducts of oxidative metabolism and moderate levels of ROS are involved in many cellular responses, including cell growth and immunity. Specifically, moderate levels of ROS can promote platelet activation and the proliferation and migration of skin cells through the activation of epidermal growth factor receptors and keratinocyte growth factor receptors, which in turn can promote wound healing [18,19]. Additionally, ROS secreted by neutrophils and macrophages can mediate the immune response by directly attacking pathogens or by activating immune-related receptors, such as the nucleotide-binding domain and leucine-rich repeat-containing family pyrin domain-containing 3 (NLRP3) inflammasome [20,21].

Under normal physiological conditions, ROS homeostasis is maintained by antioxidant defense systems, including enzymatic and non-enzymatic antioxidants [22]. Enzymatic antioxidants, such as SOD, CAT, GPX, and glutathione reductase, are the first-line antioxidant defense system that inhibits or prevents the formation of free radicals or reactive species [23]. They neutralize molecules that quickly become free radicals [24]. The second line antioxidant defense system includes non-enzymatic antioxidants, such as ascorbic acid, vitamin E, and glutathione, which rapidly deactivate oxidants such as ROS [25]. Despite this antioxidant defense system, endogenous factors such as respiratory bursts and exogenous factors such as UV irradiation, environmental pollution, and pathogens can increase ROS levels [26,27]. Increased ROS levels can lead to an imbalance between ROS and biological antioxidant levels, resulting in oxidative stress [28].

**Figure 1:**
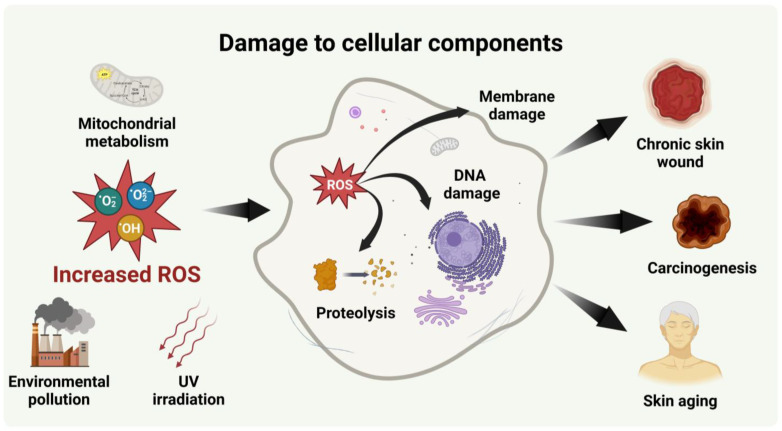
Schematic of disease development through damage to cellular components of ROS increased by various environmental stresses (created with BioRender.com).

ROS, including superoxide anion radical (O_2_^●−^) hydroxyl radical (OH^●^), and hydrogen peroxide (H_2_O_2_), are biologically produced chemically reactive molecules with high reactivity [29]. Thus, oxidative stress induced by high levels of ROS can react with cellular components, such as proteins, lipids, and DNA, leading to cytotoxicity [30]. Oxidative stress alters protein translation and increases protein susceptibility to proteolysis [31]. ROS such as OH^●^ can damage all components of DNA, including purine and pyrimidine bases and the deoxyribose backbone [32]. Additionally, oxidative stress can also induce cell death by oxidizing polyunsaturated lipids, which are abundant in cell membranes, and increasing the permeability of the cell membrane [17]. As a result of this cellular damage, oxidative stress can lead to various pathological conditions (Figure 1) [33].

### 2.2. Oxidative Stress in Chronic Skin Wound

Chronic skin wounds, an abnormal wound-healing process, are among the most common diseases caused by oxidative stress [34]. Normal wound healing, which consists of coagulation, inflammation, re-epithelialization, and remodeling, is one of the most important processes for survival [35]. However, excessive ROS generation in wound lesions caused by various environmental stresses can lead to the activation of proinflammatory genes [36,37]. This abnormal inflammatory response caused by oxidative stress in wound lesions can disrupt the transition from inflammation to re-epithelialization, leading to chronic skin wounds [38]. Once a wound becomes chronic, the prolonged inflammatory response, a key feature of chronic skin wounds, leads to the accumulation of large amounts of ROS, which in turn promotes an inflammatory response, resulting in a vicious cycle of worsening chronic skin wounds [34,39]. Moreover, excessive amounts of ROS can damage cellular components such as DNA, lipids, and proteins, and induce the hyperactivity of matrix metalloproteinases (MMPs), leading to the degradation of various extracellular matrix (ECM) proteins [40,41]. Damage to cellular components owing to oxidative stress can interfere with wound healing after the inflammatory response is resolved. As a result, excessive accumulation of ROS in wound lesions due to a variety of causes can lead to chronic wounds through an abnormal inflammatory response and can interfere with the normal wound-healing process by impeding the proliferation and migration of skin cells to fill the wound lesion.

### 2.3. Oxidative Stress in Carcinogenesis

Although cancers caused by germline mutations are rare, 90% of all cancers are thought to be caused by somatic mutations or environmental factors [42]. Several environmental stressors that cause cancer are associated with chronic inflammation. As mentioned earlier, abnormally sustained chronic inflammation can lead to a massive accumulation of ROS. This accumulation contributes to the progression of many types of cancer, including gastric, colitic, and pancreatic cancers [43].

It is generally believed that carcinogenesis begins in a single cell that becomes cancerous due to a mutation in an important gene caused by a random error in DNA replication, or due to the reaction of DNA with endogenous or exogenous chemical species, such as free radicals [44]. The most damaging free radicals to DNA are OH^●^, and many oncogenes and tumor suppressor genes can be mutated by oxidative stress caused by chronic inflammation [45]. DNA strand breaks caused by alterations in purine or pyrimidine rings are a major type of DNA damage caused by oxidative stress [46]. In particular, 8-hydroxy-2′ -deoxyguanosine (8-OHdG) is a typical oxidative DNA damage product that pairs with adenine and cytosine, leading to DNA mutation [47,48,49]. In normal cells, when cellular stresses such as DNA mutations are triggered, they eliminate themselves through cellular suicide programs such as apoptosis [50]. However, excessive ROS from various cellular stresses can cause cancer by disrupting the cell suicide program through the dysregulation of cancer-related genes [44,45]. In particular, genetic mutations caused by excessive ROS in p53, a key gene that suppresses tumor formation through apoptosis, disrupt cell death in several types of cancer, including follicular lymphoma [51]. In conclusion, chronic inflammation caused by various environmental stresses may lead to carcinogenesis via the induction of mutations in cellular components through the massive accumulation of ROS.

### 2.4. Oxidative Stress in Skin Aging

The skin is the outermost organ of the body that protects vital organs from various environmental stresses. Skin aging is divided into intrinsic aging, which is influenced by genetics and hormones, and extrinsic aging, which is caused by environmental stress [52]. Intrinsic aging inevitably occurs with age and varies considerably between individuals. It also varies by site within the same individual [53]. This intrinsic aging is due to a combination of many factors, including genetics and metabolism. ROS, which are converted from approximately 1.5–5% of the oxygen consumed by the skin, are an important factor in intrinsic aging [54]. Besides mitochondria-derived ROS from keratinocytes and fibroblasts, excessive ROS production from environmental pollutants such as UV irradiation, visible light, and ozone can cause extrinsic aging [55,56]. UV irradiation is the most common environmental stress to which the skin can be exposed and can lead to skin aging, also known as photoaging, through the overproduction of ROS [57,58]. Excessive production of ROS in the skin due to various internal or external factors leads to a decrease in the number of keratinocytes and fibroblasts, resulting in decreased epidermal turnover and collagen and proteoglycan deposition [54]. Specifically, high ROS levels in the skin stimulate activator protein-1 to block collagen synthesis and upregulate MMP expression to promote collagen degradation [59,60].

## 3. EVs Isolated from Plants

### 3.1. Characteristics of EVs

**Figure 2:**
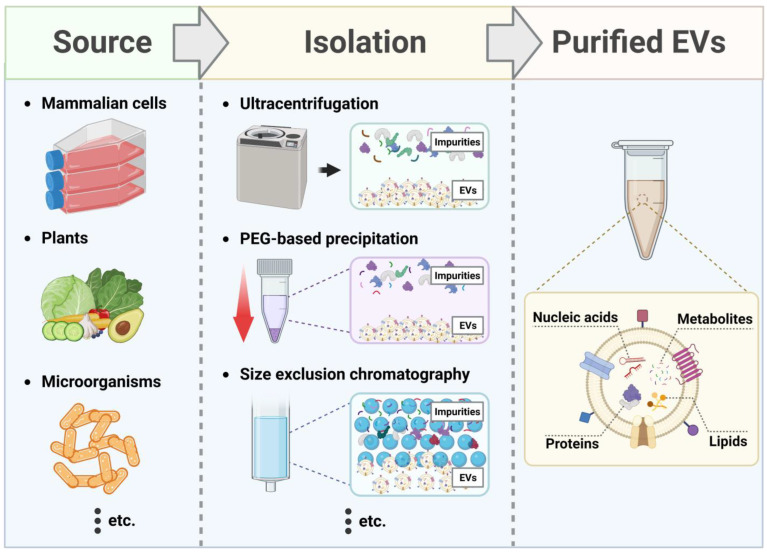
**A** schematic diagram for the isolation of EVs from various sources using different isolation methods (created with BioRender.com).

Extracellular vesicles (EVs), which consist of a lipid bilayer, are released during intercellular communication between various cell types, including animals, plants, and microorganisms (Figure 2) [61]. Based on their size and slightly different biogenesis mechanisms, EVs are classified as exosomes, microvesicles, and apoptotic bodies [62]. Exosomes, which have the smallest size (40–120 nm), initiate biosynthesis by forming early endosomes in the plasma membrane via endocytosis [62]. Early endosomes form intraluminal vesicles (ILVs) that transform into multivesicular bodies (MVBs). When MVBs fuse with the plasma membrane, exocytosis occurs, releasing ILVs into the extracellular space, and exosomes are secreted from the cell. Microvesicles belonging to another subtype of EVs are formed by budding directly outside the plasma membrane and are 100–1000 nm in size [62]. Apoptotic bodies, which have the widest range of sizes (from 50–2000 nm) are formed during apoptosis, a form of programmed cell death [62].

EVs produced via the above-mentioned biogenesis mechanism play an important role in cell-to-cell communication and the transfer of biomolecules between cells [63]. As mediators of intercellular mass transfer, EVs can act as therapeutic agents by delivering cell-derived bioactive cargo, such as nucleic acids, lipids, and proteins, which can have a beneficial effect on the biological activity of recipient cells [62]. Additionally, the EV lipid bilayer membrane structure contributes to the stable protection and delivery of their internal cargo, especially bioactive compounds [64]. EVs also can efficiently penetrate physiological barriers and tissues owing to their nanosize and intrinsic transcytosis effect. EVs also stay in the body longer owing to their prolonged circulation time [65,66,67].

Recently, several studies have reported that EVs obtained from various cell types can be used as therapeutic agents and delivery carriers owing to their biological functions and structural properties. As examples of known biological functions of EVs, mesenchymal stem cell (MSC)-derived EVs have been shown to stimulate blood vessel formation, reduce inflammatory responses, and protect against oxidative stress-induced skin damage [68,69]. In addition to the biological functions of MSC-derived EVs, cell migration- and proliferation-promoting effects of human keratinocyte-derived extracellular vesicles and anti-aging effects of human-induced pluripotent stem cell-derived exosomes have also been reported [70,71].

From a drug delivery carrier perspective, EVs are similar to liposomes, a type of synthetic lipid nanoparticle widely used to deliver nucleic acids and small molecules, in that they are also composed of phospholipid structures [72]. However, EVs have the advantage of being relatively free from safety issues because of their higher biocompatibility and lower immunogenicity and cytotoxicity compared with some synthetic liposomes [72,73]. In addition to safety, EVs have other benefits as delivery carriers. For example, unlike some synthetic liposomes, they can efficiently deliver internal cargo because they have a complex lipid composition and specific membrane proteins that may enhance endocytosis in recipient cells [74].

Taken together, EVs secreted during cell-to-cell communication of various cell types have begun attracting attention because they not only have various biological functions of delivering bioactive molecules but also show potential as drug delivery carriers because they have high biocompatibility and can stably deliver internal cargo due to their structural characteristics.

### 3.2. Characteristics of PDEVs

#### 3.2.1. Comparison of PDEVs and MDEVs

Plant-derived extracellular vesicles (PDEVs) produced from various edible plant sources, including vegetables and fruits, contain bioactive substances such as nucleic acids, proteins, lipids, and metabolites [75]. Similar to mammalian cell-derived EVs (MDEVs), PDEVs can effectively protect and deliver internal bioactive molecules through their lipid bilayer membrane structures [76]. As an example of PDEVs that can stably deliver internal cargo, Wang et al. reported that grapefruit-derived PDEVs showed higher stability than cationic DOTAP:DOPE liposomes and could stably deliver curcumin, Zymosan, and folic acid without altering their biological activity [77]. Additionally, PDEVs can be internalized by mammalian cells and act as active mediators for inter-species as well as inter-kingdom communication [78,79,80]. As an example of PDEVs-mediated interspecies communication, Mu et al. demonstrated that edible PDEVs can act as mediators of interspecies communication by inducing gene expression in mammalian cells [81].

One of the benefits of PDEVs, which have characteristics similar to those of MDEVs, is that a large number of EVs required for future therapeutic applications can be cost-effectively produced [82]. Unlike mammalian cell culture media or serum, which are required to prepare sufficient cells for MDEVs isolation, many PDEVs can be obtained using inexpensive plant sources. In fact, the volume of MSC-conditioned medium required to obtain a total of 4.03 × 10^12^ particles from MSCs was 4300 mL, which is a substantial quantity [83]. In contrast, in our previous study, the isolation of PDEVs from *Aloe saponaria* with a yield of approximately 1 × 10^10^ particles/g did not require expensive materials other than the cost of purchasing the plant [84]. Additionally, Kim et al. isolated carrot-derived EVs (CEVs), and the number of CEVs per gram of carrots was 3.24 × 10^11^ particles/g, which equaled 1.81 × 10^14^ particles/USD. These high yields of CEVs support the advantages of PDEVs in terms of large-scale production [85]. Considering that the economical large-scale production of EVs is essential for practical clinical applications, PDEVs are promising alternatives to MDEVs owing to their low production costs and time [86].

In addition to scalability for large-scale production, another unique characteristic of PDEVs compared to MDEVs is that they contain plant-specific bioactive metabolites involved in the primary and secondary metabolism of plants [87]. Wongkaewkhiaw et al. isolated *Boesenbergia rotunda* (L.) Mansf. (fingerroot)-derived PDEVs (FDEVs) and identified 58 plant-derived metabolites, including phenolic compounds such as naringenin chalcone, pinostrobin, and pinocembrin, contained in FDEVs through liquid chromatography-mass spectrometry analysis [88]. In addition, Cao et al. confirmed through electrospray ionization (ESI) scanning that *Panax ginseng*-derived EVs (PGEVs) contain ginsenoside Rg3, a triterpenoid saponin [89]. The internal cargo composition of the PDEVs revealed through these analytical methods indicates that they contain edible plant-derived metabolites that contribute to their biological activities.

Therefore, PDEVs are considered promising alternatives to MDEVs as therapeutic agents because of their biological and structural properties, low toxicity, scalability for economical large-scale production, and the advantage of containing specific chemical compounds depending on the plant of origin.

#### 3.2.2. PDEVs as Plant-Derived Antioxidants Carriers

Plants have evolved antioxidant defense systems to protect themselves from various environmental stressors that cause oxidative stress, and plants with medicinal antioxidant properties have been consumed since ancient times to treat numerous diseases. More recently, various antioxidants extracted directly from medicinal plants have been used to treat oxidative stress-related diseases [90,91]. Vitamins (particularly, vitamins C and E), phenolic compounds, and carotenoids are among the major plant-derived antioxidants used for the treatment of various diseases [13,92]. Plant-derived antioxidants can be extracted using various methods, including hydrothermal extraction, Soxhlet extraction, maceration, and sonication [93]. Silva et al. extracted phytochemicals from *Aloe aponaria*, which has anti-inflammatory and antioxidant effects, via maceration in ethanol [94,95]. HPLC analysis revealed that *Aloe aponaria*-derived extract contained flavonoids such as rutin, quercetin, and kaempferol, as well as phenolic acids such as gallic and caffeic acids, which have antioxidant properties. An *Aloe aponaria*-derived extract containing these active compounds effectively reduced protein carbonyl levels, lipid peroxidation, and non-protein thiol levels increased via H_2_O_2_ treatment in vitro, and also exhibited antioxidant effects against ultraviolet B (UVB)-induced oxidative stress in rat paws. Miyake et al. isolated the antioxidant flavonoid glycoside eriocitrin (eriodictyol 7-O-rutinoside) from a lemon peel extract prepared via hydrothermal extraction [96]. Eriocitrin has been reported to exert a protective effect against oxidative stress induced by acute exercise, in which oxygen uptake increases rapidly. Eriocitrin significantly reduced the acute exercise-induced increase in the levels of thiobarbituric acid-reactive substances, a marker of lipid peroxidation, in the liver of mice and attenuated the acute exercise-induced increase in oxidized glutathione levels [97].

These natural antioxidants are widely used because of their low toxicity, but the extraction of plant-derived antioxidants using harsh extraction methods, such as hydrothermal extraction, can lead to the degradation of heat-labile chemical molecules [98,99]. Although plant-derived phytochemicals can be extracted smoothly using appropriate extraction methods, most natural compounds are susceptible to degradation or can be metabolized into inactive derivatives in the circulation of the body [100,101]. In particular, the biological activities of phenolic compounds and ascorbic acid, which are typical plant-derived antioxidants, can vary depending on the storage and processing methods [102,103]. Furthermore, the insufficient solubility and low bioavailability of plant-derived extracts are major limitations to their application as therapeutic agents [104,105]. Curcumin, the main component of *Curcuma longa*, is one of the safest plant-derived phytochemicals with pharmacological benefits such as antioxidant and anti-inflammatory properties; however, its bioavailability is limited due to poor absorption, rapid metabolism, and rapid systemic elimination [106,107]. Moreover, resveratrol is a plant-derived phenolic compound with protective effects against cardiovascular diseases, cancer, and metabolic diseases, and has been shown to reach peak plasma concentrations of approximately 10 ng/mL 0.5 h after oral ingestion of 25 mg of resveratrol [108,109]. Therefore, less bioavailable plant extracts are required to be effective in vivo and higher doses of plant extracts are required [104].

Accordingly, drug delivery systems, such as liposomes and nanoparticles, are being applied to improve the bioavailability and stability of plant-derived extracts [110,111]. In particular, liposomes loaded with various plant-derived extracts are valuable carriers that can easily overcome the low solubility and permeability of plant-derived extracts [105]. As mentioned previously, PDEVs, which are structurally similar to liposomes, contain plant-derived metabolites, particularly antioxidants [112]. Raimondo et al. isolated *citrus limon* L. derived PDEVs (CLEVs) and characterized their internal contents using reversed-phase high-performance liquid chromatography coupled with electrospray ionization quadrupole time-of-flight mass spectrometry [113]. CLEVs were found to contain 45 compounds categorized as organic acids, flavonoids, limonoids, cinnamic acid derivatives, lysophospholipids, acyl-thioesters, carbohydrates, and phenolic acid derivatives. Flavonoids with anti-inflammatory and antioxidant effects, such as eriocitrin, quercetin, vicenin-2, naringin, hesperidin, and limonoids, such as limonin, were identified. PDEVs have also been reported to contain glutathione and ascorbic acid, which are two of the leading plant-derived antioxidants [114]. Logozzi et al. identified the presence of ascorbic acid and glutathione in mixed PDEVs using commercial detection kits. These mixed PDEVs were isolated from a variety of plant mixtures, including kiwi, orange, blood orange, lemon, papaya, and mango. In particular, antioxidants in mixed PDEVs can be protected from chemical lysis using chemical lysis buffers, and from physical lysis using ultrasound. The stability of the PDEVs in the surrounding environment was also reported by Hwang et al. [115]. Nanoparticle tracking analysis was used to confirm the stability of PDEVs isolated from *Dioscorea japonica* (YEVs) with long-term storage at −80 °C. YEVs stored long-term showed the same biological activity as fresh YEVs. Furthermore, as reported by Hwang et al., the total number of YEVs remains unchanged in acidic conditions such as the stomach, as well as in digestive enzyme-rich conditions such as the small intestine. PDEVs not only protect various bioactive substances, including antioxidants, from harsh conditions, but also successfully deliver biological molecules into various types of cells [82,116]. CLEVs isolated by Raimondo et al. were intracellularly delivered to A549 and LAMA84 cells in a time-dependent manner in vitro, and the intraperitoneally injected CLEVs accumulated in the tumor tissue 15 min, 1 h, and 24 h after injection in a mouse model of cancer [117]. Mu et al. confirmed that PDEVs isolated from grapes, grapefruits, ginger, and carrots were delivered to intestinal macrophages and stem cells via oral administration in a mouse model [81].

Taken together, it appears that PDEVs isolated from plants with antioxidant effects can not only protect the different types of antioxidants contained in them against multiple environmental stresses, but also increase their bioavailability by improving their permeability and stability.

### 3.3. Isolation Methods for PDEVs

Since EVs are used as drug delivery carriers and therapeutic agents owing to their structural characteristics and biological activities, various methods to isolate EVs are also being studied. As the structural composition of PDEVs is similar to that of MDEVs, the isolation method can be applied in the same manner. However, because the various methods currently used to isolate EVs from plant sources have different advantages and disadvantages, there is no gold standard for EVs isolation in terms of yield, purity, production time, or cost (Table 1) [86,118]. The major advantage of PDEVs over MDEVs as therapeutic agents is their ability to enable economical large-scale production, which is essential for future clinical applications. Therefore, to utilize the benefits of PDEVs, such as cost-effective scalability owing to low production costs and time, it is important to optimize an appropriate method to efficiently isolate a large number of PDEVs at a minimum cost. Therefore, in this section, we present a combination of methods used to isolate PDEVs from various plants, considering their high productivity and purity, along with the principles and characteristics of each isolation method.

#### 3.3.1. Ultracentrifugation (UC)

Differential UC (DUC) is currently the most commonly used method for isolating PDEVs from various plant sources [119,120,121,122]. First, the plants and phosphate-buffered saline (PBS) were ground together using a blender and then centrifuged at low speeds to remove bulky materials such as plant fibers and debris from the plant juice. Subsequently, the centrifugal force was progressively increased to remove smaller contaminants (other than the PDEVs) from the supernatant. In the final step, PDEVs were obtained via centrifugation at a high rate of 100,000× *g*. DUC is a representative method for EV isolation because of its relative simplicity and low potential for contamination of EVs with chemical reagents [86]. However, the high centrifugation speed of UC can damage the integrity of the structure as well as the agglomeration of EVs and requires expensive specialized equipment [123]. Additionally, a major drawback of DUC is that isolated EV samples may be contaminated because high centrifugation speeds co-precipitate lipoproteins and protein complexes that are difficult to remove [124]. Considering these problems, an isolation method combining DUC and gradient UC (GUC) has been used to increase the purity by isolating PDEVs from impurities [125,126]. GUC is a method for isolating EVs based on centrifugal force and a density gradient created by stacking solutions of different densities using sucrose or iodixanol solutions. Although EVs isolated using the combined DUC and GUC methods have higher purity, this method also requires optimization because repetitive centrifugation at a high centrifugal force results in a significant loss of EVs [123].

#### 3.3.2. Size Exclusion Chromatography (SEC)

SEC is a method for isolating EVs and biological contaminants according to different elution times because the rate at which they pass through the porous resin of the column differs depending on particle size [127]. The aggregation of EVs and structural integrity damage are not induced during the isolation process of EVs by SEC because the loaded sample passes through the column depending on gravity rather than shear force. Thus, the biological activity of the obtained EVs can be maintained [128,129,130]. Additionally, EVs isolated via SEC have high purity, but the column injection volume is limited, making it time-consuming and difficult to scale up [128]. Therefore, to improve the problem in terms of scale-up, You et al. and Kim et al. have recently reported that PDEVs with high purity and yield were effectively obtained by first concentrating plant-juice containing PDEVs through ultrafiltration to reduce the sample volume, and then isolating them via SEC [82,85].

#### 3.3.3. Polyethylene Glycol (PEG)-Based Precipitation

PEG-based precipitation is a cost-effective method for isolating PDEVs in large quantities without expensive equipment such as ultracentrifuges [131]. The PEG-based precipitation method is widely used by researchers because it uses PEG, a biocompatible polymer approved by the U.S. Food and Drug Administration (FDA) and is applied to various pharmaceuticals to reduce the solubility of EVs and to conveniently isolate them using low-speed centrifugation [132]. First, PEG-based precipitation involves a differential centrifugation process at a low speed to remove impurities such as debris from plant juices at an early stage of isolation. Then, a PEG solution of an appropriate concentration is mixed with the PDEV-containing supernatant and stored in a refrigerator, after which EVs pellets with maintained structural integrity can be obtained via low-speed centrifugation [123]. As mentioned earlier, PEG-based precipitation is simple and cost-effective; however, a major drawback is the low purity of isolated EVs obtained using this isolation method compared to others because of excess PEG or coprecipitated contaminants [123,133]. Therefore, to overcome the low purity of EVs obtained via the PEG-based precipitation method for practical applications, excess PEG and non-EV substances have been removed using additional purification processes such as UC or SEC in recent years [133,134].

## 4. Therapeutic Applications of PDEVs as Antioxidants

Oxidative stress can occur when ROS are overproduced owing to a variety of stresses and the antioxidant defense system is disrupted. Oxidative stress can damage specific tissues and contribute to various diseases. In this section, we discuss the applications of PDEVs in the reduction in oxidative stress and, more importantly, the amelioration of oxidative stress-related diseases through the reduction in oxidative stress. Table 2 summarizes the therapeutic applications of PDEVs in oxidative stress-related damage and diseases.

### 4.1. PDEVs for Oxidative Stress-Induced Damage

As mentioned earlier, PDEVs are active molecules, such as proteins, lipids, nucleotides, and metabolites that can mediate intracellular communication. In particular, PDEVs isolated from plants with antioxidant effects can resolve oxidative stress caused by various environmental stressors by directly scavenging ROS or enhancing the antioxidant defense system of recipient cells.

Among the different strawberry varieties, Romina contains many antioxidants, including anthocyanins, folic acid, flavonols, and vitamin C [142,143]. The PDEVs isolated from Romina (REVs) not only contain high vitamin C levels (416 nmol/mg EVs) but also some of the small RNAs and miRNAs found in Romina juice [135]. REVs were delivered to adipose-derived MSCs (ADMSCs) without significant toxicity and dose-dependently inhibited ADMSC apoptosis due to oxidative stress induced by H_2_O_2_ treatment. Furthermore, REVs significantly reduced the increase in ROS caused by H_2_O_2_ treatment. Similar to REVs, the presence of citrate, ascorbic acid, and short RNA in CLEVs, which are abundant in lemons, has been reported by Baldini et al. [119]. CLEVs were delivered intracellularly without cytotoxicity even after treatment with up to 50 μg of CLEVs. CLEVs protected MSCs against H_2_O_2_-induced cytotoxicity in a dose-dependent manner and the levels of H_2_O_2_-induced ROS were also significantly reduced. Additionally, the promoting effect of CLEVs on the osteogenic differentiation of MSCs without treatment with osteogenic substances has also been reported. These results are consistent with the findings that ascorbic acid and citric acid can promote the osteogenic differentiation of MSCs and demonstrate that CLEVs effectively deliver the ascorbic acid and citrate contained within the recipient cells [144,145]. Kim and Rhee have reported the antioxidant effects of CEVs [85]. CEVs were isolated at a relatively high purity of 2.28 × 10^10^ particle count/total protein μg by SEC, and no significant cytotoxicity was observed in H9C2 heart-derived cardiomyoblasts, even at concentrations 10-fold higher than the therapeutic dose of 10^11^ particles/mL. To evaluate the antioxidant effect of CEVs in H9C2 cells, oxidative stress was induced in H9C2 cells via H_2_O_2_ treatment to evaluate the antioxidant effects of CEVs. CEVs significantly inhibited the production of ROS as well as apoptosis via H_2_O_2_-induced oxidative stress. CEVs significantly inhibited the production of ROS via H_2_O_2_-induced oxidative stress and induced an increase in protein expression levels as well as mRNA gene expression levels of antioxidant-related genes such as Nrf-2, HO-1, and NQO-1. The antioxidant effects of PDEVs via the nuclear translocation of Nrf-2 have also been reported. The antioxidant effects of ginger-derived PDEVs (GEVs) via nuclear translocation of Nrf-2 have also been reported [136]. GEVs containing 6-shogaol increased the nuclear translocation of Nrf-2, and GEV treatment reduced ROS production in hepatocytes.

### 4.2. PDEVs for Oxidative Stress-Related Diseases

Oxidative stress can directly oxidize macromolecules including lipids, proteins, and DNA, leading to cytotoxicity and cell death. Because this can damage tissues or areas, the accumulation of oxidative stress is believed to be one of the causes of various diseases. Chronic skin wounds, carcinogenesis, and skin aging are among the various diseases associated with oxidative stress, and various studies are being conducted to improve these conditions through the antioxidant effects of PDEVs.

#### 4.2.1. Chronic Skin Wound

Oxidative stress from various sources at the wound site can induce an abnormal inflammatory response by activating pro-inflammatory genes, which may contribute to chronic skin wounds [36,37,38]. Additionally, oxidative stress can interfere with the proliferation and migration of skin cells to fill wound lesions by damaging cellular components and creating a proteolytic environment that is unfavorable for wound healing [40,41]. Therefore, addressing the sustained inflammatory response and creating a favorable environment for wound healing using antioxidants is one of the various strategies used for chronic skin wound healing.

**Figure 3:**
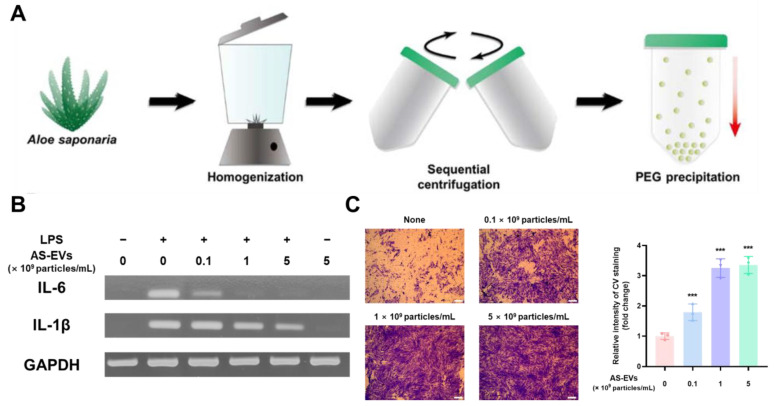
Effect of ASEVs on chronic skin wounds. (**A**) A schematic illustration of the ASEVs isolation procedure using the PEG-based precipitation method. (**B**) Effect of ASEVs on mRNA expression levels of pro-inflammatory cytokines IL-6 and IL-1b in LPS-stimulated RAW 264.7 cells. (**C**) Proliferation promoting effect of ASEVs in HDFs (*** *p* < 0.005). Adapted from Ref. [84].

Savcı et al. reported the wound-healing effect of EVs isolated from grapefruit (GFEVs) via a polymer-based precipitation method [137]. GFEVs reduced the level of intracellular ROS increased via H_2_O_2_-induced oxidative stress in HaCaT cells, a human epidermal keratinocyte cell line, to the level of the H_2_O_2_-untreated control group, as determined using the H2DCFDA assay. GFEVs with antioxidant effects promoted the proliferation and migration of HaCaT cells and increased the expression of wound healing-related mRNAs and proteins such as collagen type I, fibronectin, laminin, and vimentin. Furthermore, the human umbilical vein endothelial cells (HUVECs) tube formation assay confirmed that GFEVs promoted the formation of capillary tubes, which improved wound healing by supplying oxygen and nutrients. PDEVs isolated from *Aloe vera* (AEVs), which are widely used for various medical and cosmetic purposes, have been reported to promote wound healing through their antioxidant effects. [138]. The antioxidant activity of AEVs was evaluated using the superoxide dismutase activity assay, and the antioxidant activity of AEVs increased in a dose-dependent manner. The AEVs with antioxidant activity were not cytotoxic to HaCaT cells and were gradually internalized by HaCaT cells over 12 h via clathrin-and caveolae-mediated endocytosis and membrane fusion. Internalized AEVs in HaCaT cells reduced the ROS levels induced by H_2_O_2_ treatment in a dose-dependent manner, and 10^9^ particles/mL of AEVs exhibited antioxidant effects similar to those of quercetin, a flavonoid with potent antioxidant effects. Additionally, AEVs upregulated the mRNA expression of genes involved in antioxidant defense mechanisms (e.g., Nrf-2, HO-1, CAT, and SOD). In particular, AEVs upregulated Nrf-2, which can activate antioxidant defense mechanisms and significantly promote the migration of HaCaT cells and human dermal fibroblasts (HDFs). PDEVs isolated from *Aloe saponaria* (ASEVs), a member of the *Aloe* family, have been reported to be effective for chronic skin wound healing (Figure 3) [84]. ASEVs were not significantly toxic to RAW 264.7, HDFs, or HUVECs, which are representative in vitro model cells for chronic skin wound healing. The intracellularly delivered ASEVs effectively reduced the increased expression levels of inflammatory cytokine mRNAs such as interleukin (IL)-6 and IL-1β induced by lipopolysaccharide (LPS) treatment in the immortalized mouse macrophage cell line. ASEVs, which have anti-inflammatory effects, increased the proliferation of HDFs by up to 2.3-fold compared to untreated controls and significantly promoted the migration of HDFs. Additionally, the angiogenic effects of ASEVs, such as GFEVs, have been reported to promote the wound healing process. In conclusion, PDEVs, which have antioxidant effects that reduce inflammation, promote skin cell proliferation and migration, and exert angiogenic effects, are promising biomaterials for the treatment of chronic skin wounds.

#### 4.2.2. Carcinogenesis

Overproduction of ROS owing to various environmental stresses can induce an abnormal inflammatory response through the activation of inflammatory cytokines [34,39]. Chronic inflammation causes persistent oxidative stress through excessive ROS accumulation and can transform normal cells into cancer cells through mutations in various cellular components, particularly DNA [43]. Therefore, many researchers are attempting to resolve chronic inflammation to inhibit cancer formation. PDEVs with antioxidant effects have been recognized as natural biomaterials for resolving chronic inflammation.

**Figure 4:**
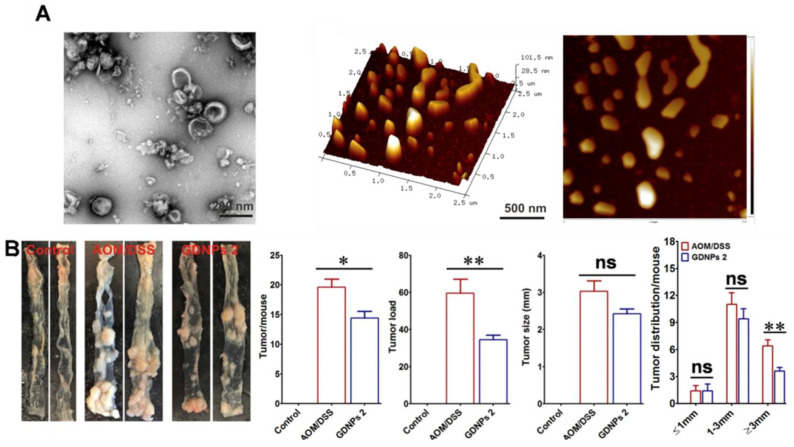
Preventive effect of GEVs on colitis-associated cancer (CAC). (**A**) Structural integrity and morphological characteristics of GEVs confirmed via transmission electron microscopy (TEM) and atomic force microscopy (AFM). (**B**) Effect of GEVs on inhibition of CAC formation. Colon tumors were obtained at the end of the CAC model mouse induction (* *p* < 0.05, ** *p* < 0.01, ns, not significant). Adapted with permission from Ref. [139]. Copyright 2016 Elsevier.

Numerous studies have reported the anti-inflammatory and antioxidant effects of ginger, which include bioactive compounds such as shogaol and gingerols [146,147]. In line with this, GEVs containing various bioactive molecules present in ginger increased the nuclear translocation of Nrf-2, which is involved in the expression of antioxidant-related genes and antioxidant genes, such as HO-1 [81,136]. Additionally, GEVs not only increase the expression of the anti-inflammatory cytokine IL-10 but also effectively inhibit the activity of the NLRP3 inflammasome, which is a key regulator of the innate immune response [120]. Zhang et al. reported the preventive effects of GEVs with antioxidant and anti-inflammatory effects on colitis-associated cancer (CAC) (Figure 4) [139]. GEVs did not show any significant toxicity at the local or systemic level despite oral administration of 0.3 mg/mouse for 7 days, having been primarily taken up by intestinal epithelial cells and macrophages. In a mouse model of colitis induced by dextran sodium sulfate (DSS) treatment, GEVs reduced the increased expression of pro-inflammatory cytokines such as TNF-α, IL-1β, and IL-6. Long-term consumption of GEVs had a protective effect against chronic inflammation in a mouse model of chronic colitis induced by knockout of IL-10. Furthermore, GEVs significantly reduced inflammation in a colorectal cancer model induced by a combination of azoxymethane (AOM) and DSS, reducing both the tumor number and tumor load per mouse. Prevention of CAC through the antioxidant and anti-inflammatory effects of tea leaf-derived PDEVs (TLEVs) has also been reported [121]. TLEVs contain polyphenols and flavones, which are found in high amounts in tea leaves, and phytochemicals with potent antioxidant and anti-inflammatory effects, such as gallic acid and EGCG. TLEVs containing various bioactive molecules inhibited LPS-induced ROS generation in RAW 264.7 and promoted the expression of antioxidant genes. Furthermore, TLEVs reduced the expression of lipopolysaccharide-induced pro-inflammatory cytokines in RAW 264.7. Through these physiological functions, TLEVs orally administered to a mouse model of DSS-induced inflammatory bowel disease restored body weight and colon length to normal levels. Furthermore, similar to GEVs, TLEVs reduced the number of tumors per mouse and the tumor size in CAC caused by persistent colitis. Collectively, these results suggest that PDEVs with antioxidant effects can be used as biomaterials to inhibit carcinogenesis by resolving abnormal chronic inflammation.

#### 4.2.3. Skin Aging

Considering that the skin is the outermost part of the body, UV irradiation is one of the most common and harmful environmental stressors [56]. UV irradiation of the skin can cause skin aging, known as photoaging, through ROS overproduction [57,58]. Therefore, PDEVs with antioxidant effects have been studied to combat the increased oxidative stress caused by UV radiation.

**Figure 5:**
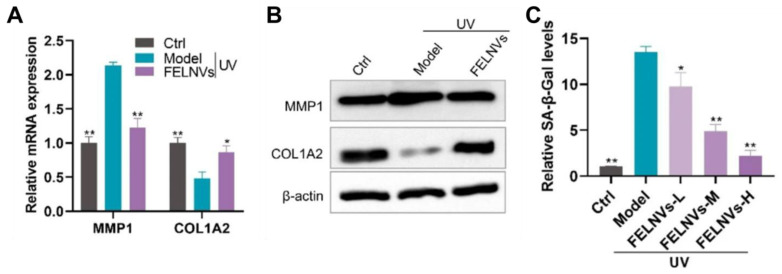
Inhibitory effect of PLEVs on UV-induced senescence in HaCaT cells. (**A**) Effect of PLEVs on mRNA expression levels of MMP-1 and COL1A2, markers of aging. (**B**) Effect of PLEVs on protein expression levels of MMP-1 and COL1A2, markers of aging. (**C**) Effect of PLEVs on SA-β-Gal levels increased by UV treatment (* *p* < 0.05, ** *p* < 0.01). Adapted from Ref. [140].

Mushrooms can be used as a source of PDEVs because they contain a variety of bioactive compounds that are part of an edible vegetable and large amounts of EVs can be isolated simultaneously [148]. Han et al. reported the inhibitory effects of PDEVs derived from *Phellinus linteus* (PLEVs), which contain compounds such as polyphenols and flavones with potent antioxidant activities, on UV-induced aging (Figure 5) [140]. PLEVs effectively inhibited UVA-induced ROS production in HaCaT cells and reduced the levels of malondialdehyde, a lipid oxide and a marker of oxidative stress. Furthermore, PLEVs increased the superoxide dismutase activity of the SOD enzyme in a dose-dependent manner. Through this antioxidant action, PLEVs restored the levels of senescence-associated-galactosidase (SA-β-Gal), a representative biomarker of skin aging, and the expression of MMP1 and COL1A2, which are markers of aging, to normal cellular levels in a UV-induced photoaging model. The anti-aging effects of PGEVs on human skin cells have recently been reported [141]. PGEVs dose-dependently reduced the level of SA-β-Gal, which was increased via replicative senescence in HDFs, and significantly reduced the mRNA expression levels of senescence markers such as p53, p21Cip1, p16INK4a, MMP1, and IL-8. PGEVs also reduced the increased levels of melanin in human epidermal melanocytes (HEM) caused by UVB-induced aging to a similar extent as Melasolv, a widely used whitening agent. Specifically, PGEVs significantly reduced the expression of tyrosinase (TYR), tyrosinase-related protein 2 (TRP2), and RAS-related protein 27 (RAB27), which are associated with melanogenesis, and reversed the morphological changes in HEMs caused by UV-induced aging to resemble normal cells. Overall, the antioxidant effects of PDEVs appear to have anti-aging effects by inhibiting the overproduction of ROS caused by environmental stresses, such as UV irradiation, and by enhancing the antioxidant defense system.

## 5. Conclusions

EVs derived from diverse sources have the capacity to transport bioactive molecules, such as nucleic acids, lipids, and proteins, to recipient cells, resulting in a range of biological functions. Recently, there has been a growing interest in understanding the composition and biological roles of EVs obtained from various edible plants. In particular, PDEVs from antioxidant-rich medicinal plants contain a variety of plant-derived antioxidants. These PDEVs are currently being investigated as natural biomaterials for the treatment of oxidative stress-induced damage and oxidative stress-related diseases, including chronic skin wound, carcinogenesis, and skin aging. Nevertheless, the current research on the antioxidant effects of PDEVs remains in its early stages. Further studies on their biological effects, as well as the development of appropriate isolation techniques, are necessary for the future clinical application of PDEVs.

## Figures and Tables

**Table 1:** Commonly used methods for isolating PDEVs.

Methods	Principles	Advantages	Disadvantages
Ultracentrifugation (UC)	Differences in sedimentation rates due to size and density of PDEVs and non-EVs components [118]	Simple operation Low chemical reagent contamination	Requirement of expensive equipment
			Damage to structural integrity
			Co-precipitation of non-vesicular proteins
Size exclusion chromatography (SEC)	Differences in column elution rate according to the size of PDEVs and biological contaminants [86]	Maintain structural integrity and biological activity	Long operation time
		Minimize co-precipitation of non-vesicular proteins	Difficult to scale up
Polyethylene glycol (PEG)-based precipitation	Reducing the solubility of PDEVs by using hydrophilic polymers for easier precipitation by low-speed centrifugation [86]	Simple operation Easy to scale up Maintain structural integrity	Co-precipitation of non-vesicular proteins
			Chemical reagent contamination

**Table 2:** Therapeutic applications of PDEVs for oxidative stress-related damages and diseases.

Application	Source of PDEVs	Isolation Method	Effects	Ref.
Oxidative stress- induced damage	Strawberry (Fruits)	Differential ultracentrifugation (DUC)	Inhibit apoptosis in a dose-dependent manner and reduce ROS levels in ADMSCs.	[135]
	*Citrus limon* L. (Fruits)	DUC	Protect MSCs against H_2_O_2_-induced cytotoxicity and reduce ROS levels.	[119]
	Carrot (Roots)	Ultrafiltration + Size exclusion chromatography (SEC)	Enhance the expression levels of antioxidant-related genes (Nrf-2, HO-1, and NQO-1) in H9C2.	[85]
	Ginger (Fruits)	Gradient ultracentrifugation (GUC)	Reduce ROS production in hepatocytes by increasing the nuclear translocation of Nrf-2.	[136]
Chronic skin wound	Grapefruit (Fruits)	Aqueous two-phase (PEG/DEX) system	Promote cell proliferation and migration of HaCaT. Increase the expression of wound healing-related genes (COL1A1, fibronectin, laminin, and vimentin). Promote the capillary tubes formation in human umbilical vein endothelial cells (HUVECs).	[137]
	*Aloe vera* (Peels)	DUC + Ultrafiltration	Promote the migration of HaCaT and human dermal fibroblasts (HDFs).	[138]
	*Aloe Saponaria* (Peels)	Polyethylene glycol (PEG)-based precipitation	Reduce the expression levels of inflammatory cytokine mRNAs (Interleukin (IL)-6 and IL-1β). Promote the migration of HDFs. Promote the capillary tubes formation in HUVECs.	[84]
Carcinogenesis	Ginger (Roots)	DUC + GUC	Increase the expression of the anti-inflammatory cytokine (IL-10). Inhibit the activity of key regulators of the innate immune response (NLRP3 inflammasome).	[120]
	Ginger (Roots)	DUC + GUC	Reduce the expression of pro-inflammatory cytokines (TNF-α, IL-1β, and IL-6) in a colitis mouse model. Protect from chronic inflammation in an IL-10 knockout mouse model. Reduce both tumor numbers and tumor loads per mouse in a colorectal cancer model.	[139]
	Tea leaf	DUC + GUC	Reduce the expression of lipopolysaccharide (LPS)-induced pro-inflammatory cytokines in RAW 264.7. Restore reduced body weight and colon length to normal levels in an inflammatory bowel disease mouse model. Reduce both tumor numbers and tumor size per mouse in colitis-associated cancer (CAC).	[121]
Skin aging	*Phellinus linteus*	DUC	Restore the levels of SA-β-Gal and the senescence markers (MMP1 and COL1A2) in UV-treated HaCaT.	[140]
	*Panax ginseng* (Roots)	GUC	Reduce the levels of senescence markers (SA-β-Gal, p53, p21Cip1, p16INK4a, MMP1, and IL-8) in aged HDFs. Reduce the protein levels of factors related to melanogenesis (melanin, TYR, TRP2, and RAB27) in UVB-treated human epidermal melanocytes (HEMs).	[141]

## Data Availability

The original contributions presented in the study are included in the article.
